# Sperm morphology and DNA fragmentation after zona pellucida selection

**DOI:** 10.1530/RAF-21-0041

**Published:** 2021-08-12

**Authors:** Rumiana Ganeva, Dimitar Parvanov, Denitsa Velikova, Magdalena Vasileva, Kristina Nikolova, Georgi Stamenov

**Affiliations:** 1 Research Department, Nadezhda Women’s Health Hospital, Sofia, Bulgaria; 2 Andrology Department, Nadezhda Women’s Health Hospital, Sofia, Bulgaria; 3 Embryology Department, Nadezhda Women’s Health Hospital, Sofia, Bulgaria; 4 Obsterics and Gynecology Department, Nadezhda Women’s Health Hospital, Sofia, Bulgaria

**Keywords:** sperm DNA fragmentation (SDF), zona adhesion, zona pellucida, sperm selection, sperm morphology

## Abstract

Sperm DNA fragmentation (SDF) and sperm morphological defects can negatively affect ART outcomes. Consequently, there is a need for additional semen processing technique that accounts for sperm DNA status and morphology prior to ICSI. The objective was to evaluate the efficacy of an additional zona pellucida adhesion-based sperm selection for obtaining sperm populations with a high percentage of normal morphology and DNA integrity as compared to native semen and routine swim-up preparation. Semen samples from 78 normozoospermic men were subjected to swim up and placed in petri dishes coated with 48 acid-solubilized zonae pellucidae. Sperm DNA fragmentation and morphology were assessed in the native semen, the swim-up samples, and the zona-adhered spermatozoa from each patient. The mean sperm DNA fragmentation of the zona-selected spermatozoa (3.5 ± 0.7%) was significantly lower than the swim-up samples (15.3 ± 5.2%) (*P*  < 0.001) and native semen (24.9 ± 7.1%) (*P*  < 0.001). All of the samples had lower levels of DNA damage after additional selection by zona pellucida adhesion. Significantly higher percentage of sperm with normal morphology was observed after zona-adhesion selection (11.4 ± 3.9%) when compared to the swim-up samples (8.9 ± 4.3%) (*P*  < 0.001) or the native semen (5.3 ± 3.2%) (*P*  < 0.001). In 94% of the samples, the percentage of spermatozoa with normal morphology increased after the additional zona selection. This study demonstrates that sperm selection by additional zona-adhesion technique yields a significantly higher percentage of spermatozoa with normal morphology as well as a significantly decreased level of DNA fragmentation when compared to the native semen and the swim-up-only prepared samples.

**Lay summary:**

High level of DNA folding known as sperm DNA fragmentation (SDF) inside each sperm and defects in the shape, size, and structure of the sperm can negatively affect assisted reproduction treatment (ART) outcomes. Consequently, there is a need for additional semen processing techniques that account for sperm quality prior to ART. Our team designed a simple technique using proteins from the coat around the egg (zona pellucida) to enhance sperm selection procedures based on natural sperm–egg interactions. Using this technique in combination with the most common techniques used in ART yields a significantly higher percentage of sperm with normal shape, size, and structure and a decreased level of DNA fragmentation. This sperm zona-selection technique would be beneficial if introduced in the ART practice to yield sperm with higher fertilization potential.

## Introduction

About 30% of infertile couples are diagnosed with idiopathic infertility due to lack of any evident pathology (Sadeghi 2015). In many such cases, though, ART failures can be attributed to the so-called ‘hidden’ male factor (Mackenna 1995). Currently, routine semen analysis, based on the evaluation of sperm count, sperm mobility, and morphology alone, provides valuable but superficial information about male fertility status. Despite being classified as normozoospermic, some men are still infertile (Wang & Swerdloff 2014).

The main characteristic used for determining the quality of the individual spermatozoa is their morphology (Gatimel *et al.* 2017). Morphological abnormalities of the sperm head have long been associated with low fertilization, implantation, and pregnancy rates (Kruger *et al.* 1988, Kahraman *et al.* 1999, Osawa *et al.* 1999). Therefore embryologists tend to select morphologically normal spermatozoa for ICSI. Recently other sperm characteristics have emerged as important markers of sperm quality, such as sperm DNA fragmentation (SDF) (Jin *et al.* 2015, Chen *et al.* 2020) and the sperm zona-adhesion ability (Hamada *et al.* 2012, Ganeva *et al.* 2019*a*).

Numerous studies have investigated the significance of sperm DNA integrity in male infertility (Cissen *et al.* 2016). It has been shown that SDF has an even higher predictive value for male fertility and reproductive outcomes than routine semen analysis (Kodama *et al.* 1997, Evenson *et al.* 1999, Zini *et al.* 2001). Some authors go as far as to claim that sperm DNA damage can have a detrimental effect on fertilization (Hamada *et al.* 2012), preimplantation embryonic development (Chi *et al.* 2017), and implantation (Jin *et al.* 2015). Moreover, high DNA fragmentation in spermatozoa used for ICSI has been related to increased incidence of miscarriage (Gil-Villa *et al.* 2010) and high rates of morbidity in the offspring (Fernandez-Gonzalez *et al.* 2008).

It is still debatable whether the conventional sperm preparation by swim-up and density-gradient centrifugation reduces the percentage of DNA fragmentation (Wang *et al.* 2014) or, on the contrary, causes the generation of reactive oxygen species impairing sperm DNA integrity (Quinn *et al.* 2018). Meanwhile, several techniques have been proposed for obtaining motile spermatozoa without DNA fragmentation such as modified hyaluronan binding (Parmegiani *et al.* 2014), microfluidic sperm sorting devices (Quinn *et al.* 2018, Parrella *et al.* 2019), and motile sperm organelle morphology examination (Bartoov *et al.* 2003, Garolla *et al.* 2014).

However, following the natural sperm selection process, another non-invasive approach has emerged (Ganeva *et al.* 2019*b*). The sperm-zona binding plays a crucial role in natural fertilization and is one of the major selective stages during the spermatozoa transition through the female tract (Liu & Baker 2000).

The ability of sperm to adhere to zona pellucida has been shown to relate to their fertilization potential and eventual IVF success (Liu *et al.* 1989, Ganeva *et al.* 2019*a*, 2019*c*). Liu and Baker (2007) showed that defective sperm-zona pellucida interaction is a significant cause of fertilization failure. The lack of zona-adhesion ability of the spermatozoa indicates lower chances of success after IVF or IUI (Liu & Baker 2000, Arslan *et al.* 2006).

It has been shown that the ability of sperm to bind to the zona correlates with the DNA fragmentation of the native semen (Liu & Baker 2007). Moreover, it has been suggested that zona-binding ability can be negatively affected by SDF (Kumar *et al.* 2013). In 2007, Liu and Baker used the hemizona technique to demonstrate that zona pellucida is highly selective for spermatozoa bearing dsDNA (Liu & Baker 2007). The selectivity of the zona pellucida for spermatozoa with normal morphology had already been proven using native zonae (Liu & Baker 1992, 1994), and the possibility of retrieving those spermatozoa for ICSI had already been discussed (Braga *et al.* 2009, Liu 2011). A number of studies have since confirmed that using zona-bound spermatozoa in ICSI improves fertilization and implantation rates and results in fewer miscarriages (Black *et al.* 2010, Liu 2011, Jin *et al.* 2016, Ganeva *et al.* 2019*c*). Compared to conventional ICSI, the use of zona-selected spermatozoa for ART has also been shown to result in improved embryo quality (Liu *et al.* 2011, Ganeva *et al.* 2019*b*).

Most of the sperm zona-selection techniques available today are still relatively complex and tedious to use in the IVF lab routine, and there is still a need for a simpler, more reliable, yet equally effective solution.

In the light of these statements, our team designed a simple technique using native zona pellucida proteins to possibly enhance sperm zona-adhesion test (Ganeva *et al.* 2019*c*) and zona-adhesion-based selection procedures. The proposed technique employs a zona protein pool obtained by acid solubilization of zonae pellucidae from healthy donors, subsequently immobilized on flat petri dishes, making it simple to use and handle, and allowing easy observation and isolation of sperm.

This study aims to evaluate the DNA status and morphology of spermatozoa selected by an addition to the conventional swim-up preparation, zona pellucida adhesion selection method and to compare them with the same patient’s native semen and swim-up-only prepared samples.

## Materials and methods

### Patients

This study includes semen samples from 78 normozoospermic male patients of a private hospital between February 2020 and March 2020 after approval of IRB (N 2/18.01.2020). Zonae pellucidae were obtained from 30 healthy donors’ germinal vesicles (GVs) after regular follicular puncture for egg donation. All included subjects signed written informed consent.

### Experimental design

In this study, sperm morphology evaluation and SDF were compared between native semen, swim-up-only prepared spermatozoa, and those subjected to double selection (swim-up and zona pellucida adhesion) in patients with normozoospermia. SDF test was conducted on frozen samples to minimize differences in the staining procedure.

Semen samples were collected by masturbation after 3–5 days of sexual abstinence. After semen liquefaction for 30 min at 37°C, an aliquot was used for regular semen analysis in accordance with World Health Organization guideline (2010) including sperm morphological evaluation according to Kruger's strict criteria, while another aliquot of native semen was frozen in sperm freezing medium (Origio, Denmark) in liquid nitrogen at –196°C for further analysis.

The remaining semen was washed in a sperm wash buffer (Origio, Denmark) by centrifugation at 600 ***g*** for 5 min and subjected to a standard swim-up preparation (15 min in 37°C at 45 degrees angle) in Global for fertilization (Life Global, Belgium) supplemented with 1% HSA v/v (Life Global, Belgium). One-half of the isolated motile spermatozoa (swim-up-only sample) were used for morphological evaluation and frozen in liquid nitrogen at –196°C for further analysis. The other half was subjected to the sperm zona-adhesion selection as described below and the zona adhered spermatozoa were also subjected to Kruger's strict morphological evaluation and frozen in liquid nitrogen at –196°C for further analysis.

### Sperm zona-adhesion selection

Sperm zona-adhesion selection was performed as previously described (Ganeva *et al.* 2019*c*). Briefly, 48 zonae pellucidae from 30 healthy donors’ germinal vesicles (GVs) were acid solubilized as each zona pellucida was placed in 5 µL drops of HCl 20% v/v for 5 min, and the solubilization was observed under light microscopy. The acid solubilized zona solutions were combined in zona pellucida protein pool, and the solution was neutralized with NaOH to pH 7.4. The protein concentration was measured by the Bradford quantification method (Bradford 1976). The zona pellucida protein pool was diluted in carbonate buffer (pH7.4) to the concentration of 50 µg/mL and 10 µL drops of the zona solution were immobilized by air drying on petri dishes. The zona pellucida coated surfaces were then covered with 10 × 10^6^ motile spermatozoa from each patient. After 30 min of incubation at RT, the petri dishes were washed from the unadhered spermatozoa. The adhered spermatozoa were detached by vigorous pipetting.

### Sperm DNA fragmentation (SDF) detection

In this study, all samples have been frozen under the same protocol so as to be stained and analyzed at the same time to avoid differences in the preparation method and the quality of the reagents used. Determination of DNA damage was performed using the halosperm G2 kit (Halotech, Spain) on frozen samples according to the manufacturer’s instructions. After thawing native semen, swim-up-only prepared spermatozoa and zona adhered spermatozoa concentrations were adjusted to 20 × 10^6^/mL. Each sample was done in duplicate and at least 300 spermatozoa on each slide were scored. Results were calculated as the percentage of sperm with fragmented DNA.

### Sperm morphology

Native semen, swim-up-only prepared spermatozoa, and zona adhered spermatozoa concentrations were adjusted to 10 × 10^6^/mL. Sperm morphology was assessed according to Kruger’s strict criteria. To minimize the counting error, each sample was done in duplicate and at least 300 spermatozoa on each slide were scored.

### Statistical analysis

Statistical analysis was performed using IBM SPSS Statistics v.21 Software, and figures were prepared using Prism version 8 by Graph Pad Software. The normality of the variables was analyzed by the Kolmogorov–Smirnov test. Data were analyzed by the Greenhouse–Geisser test. Results are shown as mean value ± s.d. and range. Using power analysis, it was calculated that at least 54 subjects were required to detect a medium effect size (0.25 s.d.) between the three groups at a significance level of 0.05 with a power of 0.80.

## Results

### Baseline characteristics of the studied patients

This study includes 78 males with semen characteristics in the normal range of reference values defined by WHO fifth edition (Table 1).

**Table 1:** Baseline characteristics of the studied patients.

	Mean ± s.d.	Range	WHO lower ref limits
Age, years	36 ± 5.4	28–51	
Semen volume, mL	3.4 ± 1.34	1.6–8.6	≥1.5
pH	7.8 ± 0.2	7.4–8.5	≥7.2
Sperm concentration, ×10^6^/mL	92.4 ± 60.7	16.3–346	≥15
Progressively motile spermatozoa, %	50.36 ± 7.73	38–71	≥32
Overall motility, %	52.9 ± 9.9	42–71	≥40
Immotile spermatozoa, %	42.6 ± 8.5	31–52	≤60

Swim-up preparation resulted in a mean sperm concentration of 177.4 × 10^6^/mL (s.d. 129.0, range: 67.3–234.1 × 10^6^/mL) with a mean number of spermatozoa 88.5 × 10^6^ (s.d. 64.5, range: 38.3–123.7 × 10^6^). After zona pellucida adhesion on average 5.0 × 10^6^ (s.d. 1.2, range: 1.2–4.7 × 10^6^), spermatozoa were obtained.

### Sperm DNA fragmentation (SDF)

**Figure 1:**
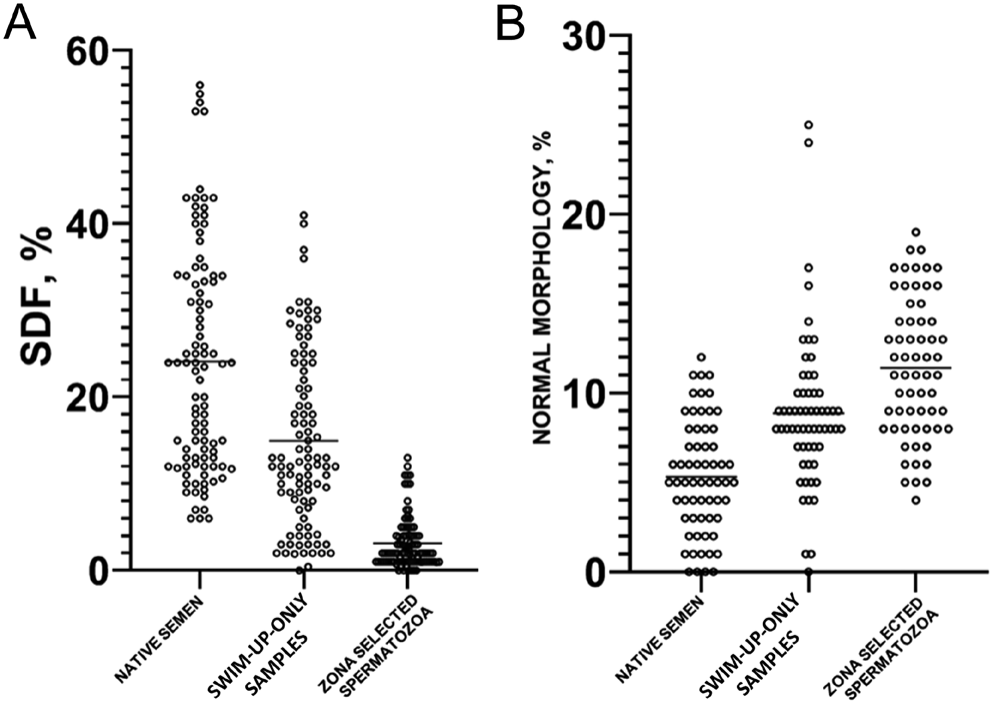
Column scatter plots of (A) sperm DNA fragmentation index (SDF) in native semen, swim-up-only samples, and zona-selected spermatozoa; (B) Percentage of morphologically normal spermatozoa in native semen, swim-up-only samples, and zona-selected spermatozoa.

In order to evaluate the effect of zona pellucida sperm selection on the SDF, we have compared the SDF of native semen, swim-up-only prepared spermatozoa, and zona adhered spermatozoa. The mean SDF in the native semen samples was 24.9% (s.d. 7.1%, range 6–56%). Statistical analysis showed that swim-up preparation reduced the percentage of DNA fragmentation significantly to 15.3% (s.d. 5.2%, range: 0–41%; *P*  < 0.001) (Fig. 1A).

**Figure 2:**
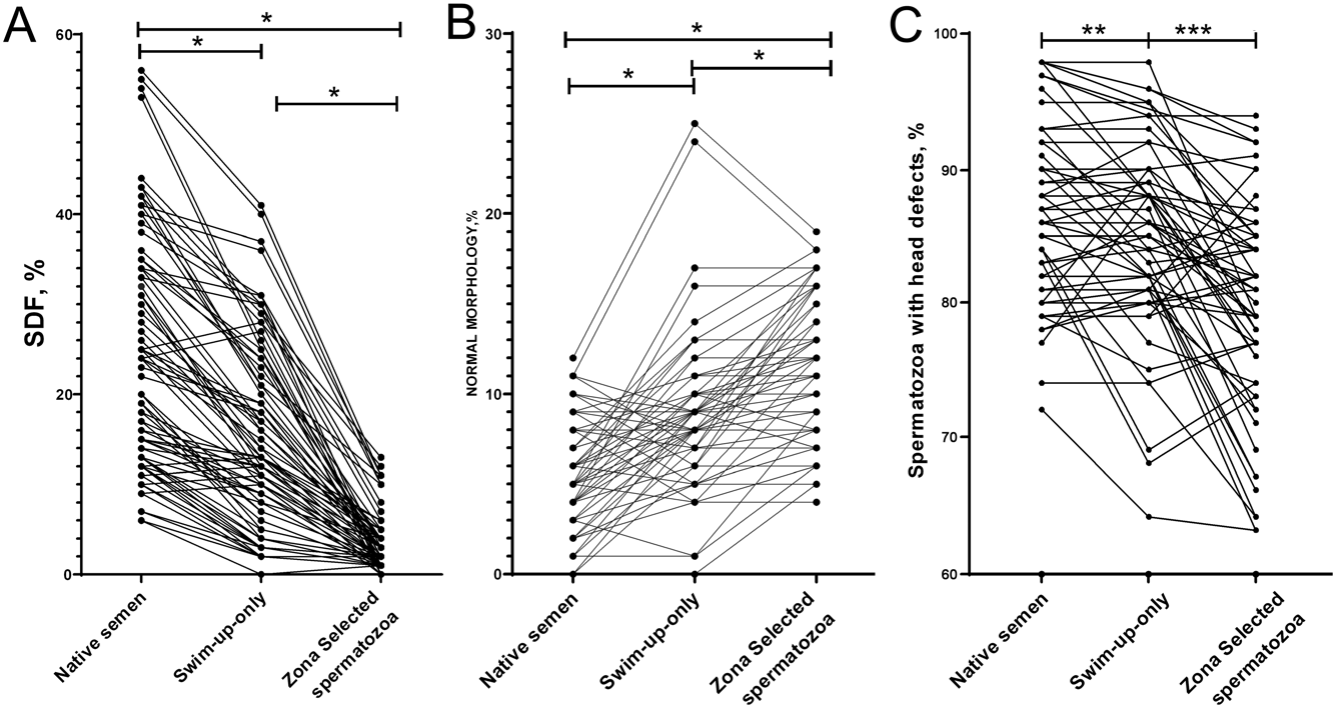
(A) Comparison of the proportion of sperm with damaged DNA in native semen, swim-up-only samples, and zona-selected spermatozoa. Each line represents an individual subject. There was a significant decrease in the SDF from native semen to swim-up-only samples (*P  < 0.001) and from swim-up-only samples to zona-selected spermatozoa (*P  < 0.001). (B) Comparison of the proportion of sperm with normal morphology in native semen, swim-up-only samples, and zona-selected spermatozoa. Each line represents an individual subject. There was a significant increase in the percentage of spermatozoa with normal morphology from native semen to swim-up-only samples (*P  < 0.001) and from swim-up-only samples to zona-selected spermatozoa (*P  < 0.001). (C) Comparison of the proportion of sperm with head defects in native semen, swim-up-only samples, and zona-selected spermatozoa. Each line represents an individual subject. There was a significant decrease in the percentage of head defects from native semen to zona-selected spermatozoa (**P  = 0.037) and from swim-up-only samples to zona-selected spermatozoa (***P  = 0.039).

**Figure 3:**
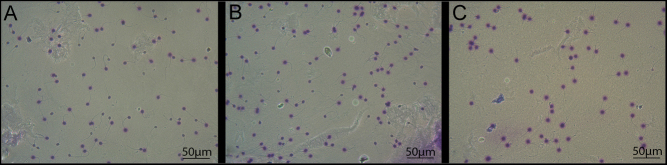
Visualization of the sperm DNA fragmentation in spermatozoa from the same subject under light-microscopy. (А) Sperm sample showing high SDF in native semen. (B) Sperm sample with lower SDF after swim-up-only. (C) Sperm sample showing the lowest SDF after the zona-selection procedure.

Moreover the additional selection through the applied zona binding procedure resulted in a significant reduction of DNA fragmentation compared to swim-up preparation (3.5 ± 0.7% vs 15.3 ± 5.2%, *P*  < 0.001, respectively). We found that in 92.3% (72/78) of the studied patients, a decrease in DNA fragmentation was observed after swim-up, but 100% (78/78) of the samples had lower DNA damage levels after additional selection by zona pellucida adhesion (Figs 2A and 3).

### Sperm morphology

To evaluate the efficacy of the combined swim-up and sperm zona-selection procedure, the morphology of the native semen, swim-up-only prepared spermatozoa, and zona adhered spermatozoa were compared. Morphologically normal spermatozoa in the native semen samples were 5.3 ± 3.2%. Swim-up preparation significantly increased the percentage of morphologically normal spermatozoa to 8.9 ± 4.3% (*P*  < 0.001) (Fig. 1B).

The additional zona-adhesion selection technique significantly increased the mean percentage of sperm with normal morphology compared to the swim-up prepared samples (11.4 ± 3.9% vs 8.9 ± 4.3%, *P*  < 0.001). Furthermore, in 94% (74/78) of the samples, the percentage of spermatozoa with normal morphology increased after the additional zona selection (Fig. 2B).

Furthermore, the three groups of morphological defects (head, midpiece, and head defects) in the native semen, swim-up-only prepared spermatozoa, and zona adhered spermatozoa were also compared. Swim-up significantly reduced the incidence of neck defects (*P*  < 0.001) and tail defects (*P*  < 0.001) in comparison to the native semen but not the percentage of head defects (*P*  = 0.155). However head defects, unlike neck and tail defects, were significantly lower after combined swim-up and zona selection in comparison to the native semen (*P*  = 0.037) and swim-up-only samples (*P*  = 0.039) (Fig. 2C).

## Discussion

A high percentage of the infertile couples are still classified as having unexplained infertility due to lack of evident pathology and male partners having a normozoospermia (Sadeghi 2015). The routine semen analysis and the current methods of sperm selection are unable to detect some important sperm characteristics such as zona-adhesion ability or DNA damage (Mackenna 1995, Wang & Swerdloff 2014).

Defective sperm-zona pellucida adhesion is one of the main causes for low fertilization rates in IVF programs (Liu & Baker 2000). Half of the patients experiencing repeated IVF failures with normal semen characteristics show disrupted sperm-zona interaction (Liu & Baker 2003). Also, high percentages of infertile couples with normozoospermic partners have a high level of SDF (Saleh *et al.* 2002). These patients are directed to ICSI rather than IVF because it has been shown that it leads to higher pregnancy rates (Li *et al.* 2006, Zhao *et al.* 2014, Zhang *et al.* 2015). Therefore couples experiencing repeated IVF failures with normal semen parameters would benefit from ICSI with additional sperm preparation techniques that would select the spermatozoa with intact DNA and membrane maturity for best chances of fertilization.

The exact role of sperm DNA fragmentation, especially in ICSI cycles, is still not fully clear. While some authors comment that increased SDF does not significantly affect ART outcomes (Sharbatoghli *et al.* 2012, Dar *et al.* 2013), other studies link DNA damage to failed IVF attempts (Osman *et al.* 2015). Nevertheless, there seems to be a consensus about the negative effect of sperm DNA damage on embryo development (Zheng *et al.* 2018) and embryo implantation success (Parikh *et al.* 2019). Some meta-analyses reveal a general trend for a high impact of SDF on the incidence of miscarriages (Robinson *et al.* 2012, Tan *et al.* 2019). Therefore, optimizing the sperm preparation before IVF to minimize the DNA fragmentation effect would be definitely beneficial for ART outcomes.

Swim-up is a ubiquitously used technique for sperm preparation preceding ART due to its effectiveness in obtaining a motile sperm population with a low percentage of apoptotic sperm (Ricci *et al.* 2009). However, previous studies have shown that routine sperm preparation and processing increase the levels of DNA fragmentation due to the excessive amounts of ROS produced during centrifugal pelleting (Jayaraman *et al.* 2012, Muratori *et al.* 2019). Consequently, there is a growing need for additional semen processing techniques that account for sperm DNA status prior to IVF (Stevanato *et al.* 2008, Jayaraman *et al.* 2012).

For this reason, we have implemented a modified and improved zona pellucida-based selection procedure. This study was designed to investigate how effectively this sperm processing technique could remove DNA-damaged and morphologically abnormal spermatozoa compared to the routinely used swim-up method.

In our study, spermatozoa were isolated based on their ability to adhere to zona pellucida proteins. This selection method has been debated for decades (Braga *et al.* 2009) and different approaches have been tried, such as hemizona assay (HZA) (Burkman *et al.* 1988, Menkveld *et al.* 1991) and the sperm-ZP binding ratio test in which intact oocytes are used (Liu *et al.* 1988). Authors applying this approach report a positive effect of zona pellucida selection on ART outcomes like fertilization rate, embryo quality, implantation rate, as well as decreased miscarriage rates (Braga *et al.* 2009, Jin *et al.* 2016). This effect was also confirmed by our previous study, which demonstrated an increase in embryo quality and higher implantation rates in couples with zona-selected sperm for ICSI (Ganeva *et al.* 2019*b*). A possible explanation for this tendency is the significantly more efficient selection of a spermatozoa population with normal morphology and lower DNA fragmentation through zona-adhesion, also observed in the present study.

The approach in this study applies a solubilized pool of homogenized zona pellucida proteins and providing a flat sperm adhesion surface. This differs from other sperm zona-binding techniques where whole or half zona with native structure is used as adhesion surface (Oehninger *et al.* 2013, Kizilay & Altay 2017). Techniques using whole or half zonae suffer from a major drawback, limiting the consistency and reliability of results due to the fact that the sperm adhesion to the intact or half zonae is affected by the zona diameter (Burkman *et al.* 1988), surface appearance (Familiari *et al.* 1988), and the thickness of the zona matrix (Franken *et al.* 1991). This undesirable sperm-binding variation between the individual zonae is avoided in the method used in this study by using a homogenized pool of dissolved zonae pellucidae from different women (Ganeva *et al.* 2019*c*). Future studies for implementing zona pellucida selection in the clinical practice would require applying homologous zonae. Using zonae pellucidae from the same patients, previous cycles could personalize the process of sperm preparation for the routine practice. Also, the method for ligand immobilization on a flat surface was proven to be an efficient technique in other sperm isolation procedures as it allows for easy observation of the adhered spermatozoa and their subsequent selection for IVF or ICSI (Huszar *et al.* 2003, Szucs *et al.* 2015).

Both sperm preparation methods employed in the present study (swim-up and zona pellucida selection) yielded sperm populations with significantly lower DNA damage than the native, unprocessed fraction. Sperm preparation through combined swim-up and zona adhesion resulted in a substantially increased percentage of spermatozoa with intact chromatin compared to the conventional swim-up, which could improve the chance of achieving a successful pregnancy and live birth after ART treatment. The positive effect of the combined swim-up and zona-selection procedure is due to the selection of motile spermatozoa (by swim-up) and andthe additional selection of functionally mature spermatozoa (by zona-adhesion selection). The immature spermatozoa bearing highly fragmented DNA and the morphologically abnormal spermatozoa are unable to pass these two stages of selection. These results are in agreement with previous studies that apply a similar procedure for zona pellucida sperm selection. In 2007, Liu and Baker concluded that zona pellucida is highly selective for spermatozoa bearing dsDNA. Other authors report not only minimal DNA fragmentation but also a lower frequency of chromosomal aneuploidies of both hyaluronan-bound and zona pellucida-bound spermatozoa (Yagci *et al.* 2010). In addition, our results demonstrate that spermatozoa that successfully adhered to the solubilized zona pellucida pool have better morphology. The selectivity of the zona pellucida for spermatozoa with normal morphology has been already demonstrated by other authors using native zonae (Liu & Baker 1992, 1994). Therefore it can be suggested that zona pellucida is a sensing barrier for spermatozoa bearing both disrupted DNA and morphological modifications. The molecular mechanisms behind this selective stage of fertilization are still unclear. However, studies have shown an inherent relation between SDF and poor sperm morphology, particularly sperm head defects (Skowronek *et al.* 2012, Jakubik-Uljasz *et al.* 2020).

Furthermore, it has been shown that spermatozoa attached to the zona pellucida possess specific morphological characteristics, particularly in the acrosomal region (Liu & Baker 1992, Garrett *et al.* 1997). Acrosomal abnormalities in otherwise morphologically normal sperm have been shown to correlate with an impaired zona-binding ability (Liu & Baker 1992, Menkveld *et al.* 1996), and even sperm with round heads and missing acrosome do not bind to the zona pellucida at all (Von Bernhardi *et al.* 1990, Bourne *et al.* 1995). Also, zona pellucida seems to have specific sperm morphometric preferences (Garrett *et al.* 1997), which was further confirmed by the fact that a defect in the zona-binding protein gene (zpbp1) in men leads to sperm head morphological anomalies and no zona binding (Yatsenko *et al.* 2012). Mice lacking *Zpbp1* have defective sperm head morphology with characteristics reminiscent of teratozoospermia in infertile men due to the increased number of head abnormalities (Lin *et al.* 2007). These results were confirmed in our study, which indicates that zona adhered spermatozoa have a significantly lower percentage of head abnormalities than the native semen and the swim-up-only samples. Even though numerous studies have confirmed the selectivity of human zona pellucida for sperm with normal morphology, some authors found that sperm with specific morphological abnormalities can still successfully bind to the zona (Liu *et al.* 1988, Garrett *et al.* 1997). In our study, it is also shown that the percentage of the overall midpiece and tail abnormalities did not differ significantly between the zona-selected spermatozoa and the native semen.

While it is true that ICSI itself can bypass many sperm abnormalities like reduced motility and severe morphological defects, an additional selection step based on the physiological ability of spermatozoa to bind to zona pellucida can be used to improve results after ICSI further.

This approach could also be applied in cases with poor semen characteristics. Sperm-zona binding test has been successfully applied in a group of patients with suboptimal semen characteristics (Franken *et al.* 1990). However, detailed information on the sperm-zona-adhesion properties in those men is missing. In teratozoospermic patients, the application of the combined swim-up and zona selection would help to isolate the spermatozoa with normal morphology. In cases such as asthenozoospermia, the yield of motile spermatozoa after swim-up would be very low. Further selection by zona-adhesion would result in much less spermatozoa compared to the samples used in this study. Thereby, the small percentage of sperm cells having high motility, membrane maturity, low SDF, and normal morphology would be easily distinguished from the whole sperm population. Therefore, future studies are needed to analyze the efficacy of the sperm zona-adhesion selection in patients with teratozoospermia and asthenozoospermia.

Therefore, in patients experiencing repeated IVF failures, an additional zona-selection step prior ICSI can be considered. In this way, we could avoid unpredicted IVF failure caused by one of the ‘hidden’ male factors associated with the membrane maturity of the sperm.

## Conclusion

This study demonstrates that sperm selection by combined swim-up and zona-adhesion technique yields a significantly higher percentage of spermatozoa with normal morphology as well as a decreased level of DNA fragmentation when compared to the native semen and the swim-up-only prepared samples. The optimization of the sperm zona-selection technique would be beneficial if introduced in cases of ‘hidden’ male factor, in which normozoospermic men face reproductive failure due to underlying sperm DNA damage.

## Declaration of interest

The authors declare that there is no conflict of interest that could be perceived as prejudicing the impartiality of the research reported.

## Funding

This work did not receive any specific grant from any funding agency in the public, commercial, or not-for-profit sector.

## Human rights statement and informed consent

All the procedures were followed in accordance with the ethical standards of the responsible committees on human experimentation (institutional and national) and in accordance with the Helsinki Declaration of 1964 and its later amendments. Informed consent was obtained from all the patients to be included in the study.

## Animal studies

This article does not contain any study with animal participants that were performed by any of the authors.

## Author contribution statement

R G, D P and G S contributed to study design. R G, N K, M V and D V contributed to data acquisition. R G and D P contributed to data analysis. All authors contributed to interpretation of data. R G and D P contributed to draft the paper or revised it critically. All authors have contributed to critical discussion and reviewed the final version of the article and approve it for publication.
